# Psychometric study of the Persian short-form eight-item Parkinson’s disease questionnaire (PDQ-8) to evaluate health related quality of life (HRQoL)

**DOI:** 10.1186/1477-7525-12-78

**Published:** 2014-05-20

**Authors:** Seyed-Mohammad Fereshtehnejad, Nader Naderi, Arash Rahmani, Gholam Ali Shahidi, Ahmad Delbari, Johan Lökk

**Affiliations:** 1 Division of Clinical geriatrics, Department of Neurobiology, Care Sciences, and Society (NVS), Karolinska Institutet, Stockholm, Sweden; 2 Firoozgar Clinical Research Development Center (FCRDC), Firoozgar Hospital, Iran University of Medical Sciences, Tehran, Iran; 3 Medical Student Research Committee (MSRC), Faculty of Medicine, Iran University of Medical Sciences, Tehran, Iran; 4 Movement Disorders Clinic, Department of Neurology, Faculty of Medicine, Iran University of Medical Sciences, Tehran, Iran; 5 Iranian Research Center on Aging, University of Social Welfare and Rehabilitation, Tehran, Iran; 6 Department of Geriatric Medicine, Karolinska University Hospital, Stockholm, Sweden; 7 Karolinska Institutet, Department of Neurobiology, Care Sciences and Society (NVS), Division of Clinical Geriatrics, Novum 5th floor, Stockholm 14186, Sweden; 8 Mental Health Research Center, Tehran Psychiatry Institute, Iran University of Medical Sciences, Tehran, Iran

**Keywords:** Parkinson’s disease, Parkinson’s disease questionnaire (PDQ), Health-related quality of life (HRQoL), Reliability, Validity, Psychometric properties

## Abstract

**Background:**

To assess validation and reliability of the Persian version of the short-form 8-item Parkinson’s disease questionnaire (PDQ-8) and to compare its psychometric properties with that of the long-form questionnaire (PDQ-39) in order to evaluate the health-related quality of life (HRQoL) in patients with Parkinson’s disease (PD).

**Methods:**

This cross-sectional study was conducted on 114 non-demented idiopathic PD (IPD) patients consecutively recruited from an outpatient referral movement disorder clinic. Patients were interviewed to fill in the Persian version of PDQ-39 and PDQ-8 questionnaires and clinical examination was performed to measure disease severity indices.

**Results:**

The Cronbach’s alpha coefficient of the entire PDQ-8 was 0.740 (95% CI: 0.661-0.806). Replacement of PDQ-8 items with other questions with the highest internal consistency within each dimension of the original PDQ-39 did not improve Cronbach’s alpha coefficient [0.723 (95% CI: 0.639-0.794)]. The scores from both PDQ-8 and PDQ-39 had significant correlation with the Hoehn & Yahr (r_PDQ-8_ = 0.376, r_PDQ-39_ = 0.442), and Schwab & England (r_PDQ-8_ = -0.503, r_PDQ-39_ = -0.598) disease severity scales and disease duration (r_PDQ-8_ = 0.342, r_PDQ-39_ = 0.396).

**Conclusions:**

Persian version of the short-form PDQ (PDQ-8) was shown to be a valid and reliable instrument to assess disease-specific HRQoL in a PD population when used independently. Although the PDQ-8 items were not necessarily those with the highest internal consistency in the components of PDQ-39, they entirely showed proper psychometric properties especially in mental and behavioral aspects. PDQ-8 is a practical and informative instrument in daily clinical practice where clinicians are in shortage of time and when a validated self-reported brief questionnaire is of value.

## Introduction

Assessment of health related quality of life (HRQoL) is of great importance in chronic conditions [1]. Parkinson’s disease (PD) is a chronic neurodegenerative disorder that presents with motor dysfunctions as tremor, balance impairment, gait and postural problems, rigidity and bradykinesia. In addition, a wide range of non-motor symptoms including sleep behavior disorder, cognitive, emotional, personality changes, sensorial and autonomic disturbances also occur [2]. Altogether, these symptoms could considerably influence patients’ HRQoL. Self-reporter HRQoL instruments evaluate the impact of these factors on patient’s daily life better than other measures used by clinicians during the routine evaluation of PD patients.

Until now, the PDQ-39 has been introduced as the most valid standardized instrument to measure HRQoL in PD populations [3]. This questionnaire includes eight different domains and has been translated and validated into more than 40 languages [4-6]. A short-form version has been developed with 8 items, the PDQ-8, which consists of only one selected item from each of the eight dimensions in the original PDQ-39 questionnaire [7,8]. Although PDQ-8 is thought to be easily implemented, more feasible and less time-consuming compared to the original version [9,10], there are few evidences to compare psychometric properties of these two formats in different cultures and/or languages.

Regarding the inevitable role of cultural barriers on validity of psychological instruments, it seems necessary to reevaluate the shortly formatted scales such as the PDQ-8. The Persian translation of the PDQ-39 has previously been validated [11]. However, there is no study on the appropriateness and precision of the short-form version. The purpose of this study was: 1) to assess the validity and reliability of the Persian version of the short-form 8-item PDQ (PDQ-8); and 2) to compare the psychometric properties of the Persian-translated short- versus long-form versions of the questionnaire to evaluate the HRQoL in PD patients.

## Methods

### Study setting & ethical considerations

This cross-sectional study was conducted in a referral movement disorder clinic in Tehran, Iran during 2011-2012. A total number of 114 Iranian PD patients was enrolled in this study and filled in the Persian version of the PDQ-39 and PDQ-8 questionnaires. The study protocol was approved by the research committee of the Firoozgar Clinical Research Development Center (FCRDC) affiliated to Iran University of Medical Sciences. This study was a collaborative project between FCRDC in Tehran, Iran and Karolinska Institutet in Stockholm, Sweden. Patients were verbally informed about the aims of the study prior to the enrollment. In case of disagreement, no extra evaluation was performed in addition to his/her routine work-up in the clinic. All collected data was stored and treated according to the ethical guidelines of medical research and the identity of research participants was protected.

### Participants

Diagnosis of idiopathic Parkinson’s disease (IPD) was made by a neurologist specialized in movement disorders using the United Kingdom Brain Bank criteria [12] for all of the participants. Other eligible criteria consisted of age ≥ 30 years, acceptable cognitive status based on the mini-mental state examination [(MMSE) > 24] [13] and not having the signs of atypical parkinsonism such as the multiple system atrophy (MSA), progressive supranuclear palsy (PSP), vascular or drug-induced parkinsonism.

### Data collection

Data collection was performed through face-to-face interviews with the patients. A group of trained medical students and general physicians performed the interviews to fill in the main study questionnaires and baseline checklist. A movement disorder specialist did all of the clinical examinations and filled in the PD-related scales. A demographic checklist consisted of baseline variables (age and sex), level of education, co-morbidities, duration of PD (time passed from diagnosis) and history of levodopa administration. Clinical characteristics of PD was assessed using the Unified Parkinson's Disease Rating Scale (UPDRS) [14], Hoehn & Yahr stage [15] and Schwab & England activity of daily living (ADL) scale [16] during “on” status.

As the most commonly used scale in clinical studies of PD [17], UPDRS was used to evaluate the severity of PD covering different aspects including mentation, behavior, and mood (part I), activities of daily living (ADL) (part II), motor examination (part III) and treatment complications (part IV). The UPDRS has a total of 147 points and higher scores reflect worse disability [14]. The Hoehn and Yahr stage is another widely used clinical rating scale defining broad categories of motor function in PD. It evaluates the severity of PD based on functional disability and clinical findings. It contains 5 stages, where 0 indicates no visible symptoms of PD, and 5 shows symptoms on both sides of the body representing the PD patients who are unable to walk. Therefore, a higher stage shows greater levels of functional disability [15]. The Schwab and England ADL scale is another global instrument for assessing the ability to perform daily activities in terms of speed and independence adopted for PD patients. A score of 100% indicates total independence, falling to 0% showing a state of complete dependence in bed-ridden individuals. Therefore, higher scores show greater level of independence in ADL [16]. In addition to PD-related scales, the Persian-translated short- and long-form versions of the Parkinson’s disease questionnaire (PDQ-8 and PDQ-39) were used to evaluate the HRQoL.

### Long-form 39-item Parkinson’s disease questionnaire (PDQ-39)

The PDQ is the most commonly used instrument measuring HRQoL in PD patients. The original long-form questionnaire contains 39 items assessing eight different domains of HRQoL in PD: mobility (10 questions), activities of daily living (ADL) (6 questions), emotional well-being (6 questions), stigma (4 questions), social support (3 questions), cognitions (4 questions), communication (3 questions) and bodily discomfort (3 questions). All questions of the PDQ-39 are answered through a Likert-scale ranging from 0 to 4 where 0 = never, 1 = occasionally, 2 = sometimes, 3 = often and 4 = always. Based on the number of items and the maximum possible score for each domain, the score is calculated as a scale ranging from 0 to 100 where 0 shows no problem at all and 100 represents the maximum level of problem in that specific dimension of HRQoL. Consequently, the total score of the PDQ-39 is calculated as the mean score of all eight dimensions [18]. In this study, we used the Persian-translated version of the PDQ-39 questionnaire, which has previously been shown to have a high reliability with a Cronbach’s alpha coefficient of 0.93 for the total summary index. The validity of the Persian-translated version of the PDQ-39 was also confirmed by forward and backward translation method in the previously published report [11].

### Short-form eight-item Parkinson’s disease questionnaire (PDQ-8)

In 1997, Jenkinson et al developed a short-form version of the PDQ-39 with eight items (PDQ-8), which only had one selected question from each of the eight dimensions (questions 7, 12, 17, 25, 27, 31, 35 and 37) of the original PDQ-39 questionnaire [8]. While filling in the original PDQ-39 questionnaire, the eight items of the PDQ-8 are already nested. However, in addition to the nested PDQ-8 in the PDQ-39 questionnaire, we also independently used the PDQ-8 questions at the same visit. Then, it became possible to compare the results of the nested and independent PDQ-8 questionnaires.

### Statistical analyses

All data from the baseline checklist and the main questionnaires were entered into the SPSS software version 20 (IBM; Chicago, IL, USA). In all analytical procedures, a two-sided *P*-value <0.05 was considered as the statistical significant level to reject the underlying null hypothesis.

#### I. Description

Continuous and discrete numerical variables were described using the mean and standard deviation (SD), whereas, the relative frequency percentage was used to describe nominal and categorical variables. In order to guarantee the acceptability of the PDQ-39 and PDQ-8 scales, floor and ceiling effects were calculated to report the relative frequency of extreme answers to the items, which should be less than 15% [19].

#### II. Exploratory factor analysis

In order to assess the unidimensionality of the entire PDQ-8 questionnaire, exploratory principal factor analysis was performed. Based on the Kaiser rule, an Eigen value of greater than 1 was considered to indicate the best-fitted structure for the scale. However, the tendency to over-extract the number of factors was also taken into account [20].

#### III. Reliability

Internal consistency was assessed using Spearman correlation test where the mean score of each item was correlated with the sum of either PDQ-39 or PDQ-8 score. Cronbach’s alpha coefficient and the 95% confidence interval (CI) of the point estimations were calculated for the entire questionnaire for both the nested and independent PDQ-8 scales. Furthermore, the intraclass correlation coefficient (ICC) was calculated to assess the inter-rater reliability between the entire score of the independent and nested PDQ-8 questionnaires. In the other words, the two raters in this design were the two versions of the PDQ-8. One sample *T* test was used to check if the difference between the scores of independent and nested PDQ-8 questionnaires were statistically different from the value 0. Afterwards, the corresponding Bland-Altman plot with the 95% limits of agreement was generated.

#### IV. Validity

Spearman correlation test was used to evaluate criterion validity of the total score of the PDQ-8 and PDQ-39 questionnaires in relation with the baseline and PD-associated variables. The underlying hypothesis was to check if the instruments are valid enough to show the changes in HRQoL in relation to the changes in variables that are expected to affect HRQoL in PD patients. For this purpose, we hypothesized that the HRQoL become poorer (higher PDQ-8 and PDQ-39 scores) with increasing severity of PD showed by a higher Hoehn & Yahr stage, a lower Schwab & England ADL scale, a higher UPDRS score and daily dose of levodopa. In addition, a longer duration of disease and an older age were also supposed to accompany with a worse HRQoL score.

## Results

### Baseline characteristics

The mean age of the study population at the time of enrollment was 61.3 (SD = 11.0) *yrs* ranging between 38 and 91. Over three-quarter (78.1%) of the participants were male and the mean duration of PD was 6.3 (SD = 5.1) *yrs*. With respect to the severity of PD, the majority of patients (69.3%) were in the stage 2 or less in the Hoehn & Yahr scale. Other baseline and disease-related characteristics of the study samples are summarized in Table 1.

**Table 1:** Baseline, clinical and Socio-demographic characteristics of the recruited Parkinson’s disease patients (n = 114)

**Characteristics**	**Value**
**Age***(yr)*	
Mean (SD)	61.3 (11.0)
**Gender***NO. (%)*	
Female	25 (21.9)
Male	89 (78.1)
**Level of Education***NO. (%)*	
Illiterate	4 (3.5)
Primary and/or Secondary	32 (28.3)
High School/Diploma	34 (30.1)
College and/or University	43 (38.1)
**Duration of Disease***(yr)*	
Mean (SD)	6.3 (5.1)
**Co-morbidities***NO. (%)*	
Depression	25 (22.3)
Cardiovascular Disease	19 (17.1)
Hypertension	18 (16.1)
Diabetes	15 (13.5)
Osteoarthritis	10 (9.0)
**UPDRS Score**	
Mean (SD)	1.8 (2.0)
Part I-*mental*	11.3 (7.7)
Part II-ADL	14.7 (9.6)
Part III-motor	3.4 (2.8)
Part IV-complications	31.3 (18.1)
Total	
**Hoehn & Yahr Stage**	
Mean (SD)	1.9 (0.9)
**Schwab and England activities of daily living score***(%)*	
Mean (SD)	81.7 (17.7)
**Daily levodopa dose***(mg)*	
Mean (SD)	850 (495)
**Duration of levodopa administration***(yr)*	
Mean (SD)	4.6 (4.5)

### Exploratory factor analysis

A one-factor solution seemed to be the best fitted model to explain the variance of the PDQ-8 scores. The first component had an eigenvalue of 2.98 and explained 37.31% of the variance while the second component only represented 16.59% of the variance in the PDQ-8 questionnaire. There was neither floor nor ceiling effect in the answers to the items of the questionnaires.

### Internal consistency

Table 2 shows the results for reliability analyses of each of the eight scales in the PDQ-39 and the corresponding items of PDQ-8 embedded within the PDQ-39 questionnaire as well as the eight items of the independent PDQ-8 questionnaire. The total Cronbach’s alpha coefficient for the entire PDQ-39 was calculated as 0.939 (95% CI: 0.922-0.954, *P* < 0.001). The “*mobility*” and “*activity of daily living (ADL)*” scales showed the largest Cronbach’s alpha coefficients while the lowest reliability was seen in the “*bodily discomfort*” domain.

**Table 2:** Spearman and Cronbach’s alpha correlation of the items of PDQ-39 and PDQ-8 for item-to-scale internal consistency in Iranian Parkinson’s disease patients (n = 114)

**Scale**	**Item**	**Spearman Rho**	**Corrected correlation**	***Cronbach's α if item deleted***
**Mobility**	Q1	0.648	0.674	0.916
	Q2	0.722	0.743	0.913
	Q3	0.818	0.777	0.910
	Q4	0.842	0.773	0.910
	Q5	0.719	0.677	0.915
	Q6	0.719	0.649	0.917
	Q7*	0.768	0.757	0.911
	Q8	0.742	0.767	0.910
	Q9	0.606	0.566	0.921
	Q10	0.704	0.672	0.916
	**PDQ-8-1**	0.832	-	Total = 0.922 (95% CI: 0.899-0.942)
**Activity of daily living (ADL)**	Q11	0.748	0.773	0.858
	Q12*	0.814	0.804	0.852
	Q13	0.857	0.767	0.858
	Q14	0.675	0.474	0.906
	Q15	0.738	0.707	0.867
	Q16	0.697	0.740	0.863
	**PDQ-8-2**	0.763	-	Total = 0.888 (95% CI: 0.852-0.917)
**Emotional well being**	Q17*	0.764	0.665	0.820
	Q18	0.618	0.587	0.834
	Q19	0.738	0.736	0.807
	Q20	0.604	0.512	0.848
	Q21	0.800	0.695	0.813
	Q22	0.764	0.623	0.828
	**PDQ-8-3**	0.758	-	Total = 0.850 (95% CI: 0.803-0.889)
**Stigma**	Q23	0.815	0.717	0.821
	Q24	0.705	0.530	0.891
	Q25*	0.857	0.801	0.787
	Q26	0.874	0.803	0.783
	**PDQ-8-8**	0.831	-	Total = 0.862 (95% CI: 0.815-0.899)
**Social support**	Q27*	0.853	0.408	0.745
	Q28	0.660	0.543	0.505
	Q29	0.566	0.598	0.521
	**PDQ-8-4**	0.680	-	Total = 0.675 (95% CI: 0.556-0.766)
**Cognition**	Q30	0.645	0.334	0.696
	Q31*	0.689	0.575	0.540
	Q32	0.699	0.568	0.542
	Q33	0.680	0.393	0.656
	**PDQ-8-5**	0.668	-	Total = 0.677 (95% CI: 0.568-0.764)
**Communication**	Q34	0.833	0.424	0.691
	Q35*	0.727	0.579	0.413
	Q36	0.584	0.518	0.590
	**PDQ-8-6**	0.649	-	Total = 0.662 (95% CI: 0.539-0.757)
**Bodily discomfort**	Q37*	0.809	0.520	0.458
	Q38	0.813	0.477	0.524
	Q39	0.583	0.383	0.640
	**PDQ-7**	0.806	-	Total = 0.646 (95% CI: 0.516-0.745)

*Embedded items of PDQ-8 in PDQ-39.

All correlations were statistically significant with *P* < 0.001.

All of the PDQ-39 items (including the representative items of the PDQ-8) had statistically significant correlations with the scale-specific total score (all *P* < 0.001). The Q26 showed the highest inter-scale Spearman correlation coefficient and the Q27 was the only representative item of PDQ-8 to have the largest inter-scale correlation coefficient of PDQ-39. However, the items Q12 (PDQ-8-2), Q31 (PDQ-8-5), Q35 (PDQ-8-6) and Q37 (PDQ-8-7) that correspond to the items of PDQ-8 represented the highest corrected correlation coefficients within their specific scales.

Table 3 summarizes the results for reliability analyses of the independent PDQ-8 questionnaire in Iranian PD patients. Total Cronbach’s alpha coefficient of the entire PDQ-8 was 0.740 (95% CI: 0.661-0.806, *P* < 0.001). The item PDQ-8-3 (“*emotional well being*”) showed the largest Spearman and corrected coefficients in correlation to the total score of the PDQ-8. In addition, deletion of this item (PDQ-8-3) represented the largest statistical contribution to the decrease of the Cronbach’s alpha of the entire PDQ-8. By contrast, the item PDQ-8-2 that represents “ADL” had the lowest corrected correlation coefficient and the lowest contribution of the total Cronbach’s alpha coefficient of the entire PDQ-8.

**Table 3:** Spearman correlation and Cronbach’s alpha of the items of independent PDQ-8 for item-to-scale internal consistency in Iranian Parkinson’s disease patients (n = 114)

**Item**	**Spearman Rho**	**Corrected Correlation**	***Cronbach's α if Item Deleted***
**PDQ-8-Q1**	0.688	0.457	0.710
**PDQ-8-Q2**	0.547	0.349	0.740
**PDQ-8-Q3**	0.697	0.541	0.691
**PDQ-8-Q4**	0.494	0.483	0.708
**PDQ-8-Q5**	0.462	0.401	0.720
**PDQ-8-Q6**	0.506	0.486	0.707
**PDQ-8-Q7**	0.623	0.523	0.697
**PDQ-8-Q8**	0.468	0.330	0.733

All internal consistency correlations are statistically significant with *P* < 0.001.

**Figure 1:**
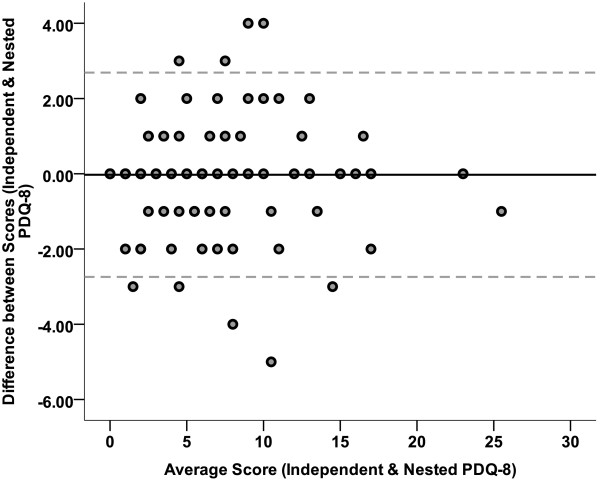
Bland-Altman plot of the scores from independent and nested PDQ-8 showing the mean difference of -0.026 (solid line) and the 95% limits of agreement of -2.742 to +2.689 (dotted lines) for the difference between the two scores.

Further analysis was performed to check if the replacement of PDQ-8 items with other questions could improve the internal consistency of the scale. For this purpose, the items showing the highest internal consistency within each dimension of the original PDQ-39 (Table 2) were selected consisting of Q4 (mobility), Q13 (ADL), Q21 (emotional well-being), Q26 (stigma), Q27 (social support), Q32 (cognition), Q34 (communication), and Q38 (bodily discomfort). For these new eight items, the Cronbach’s alpha coefficient was calculated as 0.723 (95% CI: 0.639-0.794). An ICC of 0.983 (95% CI: 0.976-0.989) was found for the inter-rater reliability between the independent and nested versions of the PDQ-8. As shown by the Bland-Altman plot (Figure 1), the mean difference between the entire independent and nested PDQ-8 questionnaires was not significantly different from the value of 0 [-0.026 (SD = 1.39), *P* = 0.840].

### Criterion validity

In order to assess and compare the criterion validity of the PDQ-8 and PDQ-39 questionnaires, the total scores of these instruments were correlated with some baseline and disease-related variables (Table 4). Except for age, all other variables were significantly correlated with the scores of both PDQ-8 and PDQ-39 questionnaires (all *P* < 0.05). There was a significant direct correlation between the total score of UPDRS and PDQ-8 and PDQ-39 questionnaires. Regarding different domains of the UPDRS scale, the PDQ-8 showed a larger coefficient in correlation with the mental (part-I) and complications parts (part IV) compared to the PDQ-39 while in other domains, the PDQ-39 had a larger correlation coefficient (Table 4). The scores from both questionnaires, PDQ-8 and PDQ-39 also had significant correlation with the Hoehn & Yahr and Schwab & England disease severity scales as well as with disease duration.

**Table 4:** Spearman correlation of the total score of PDQ-39 and PDQ-8 questionnaires to evaluate the convergent validity in association with the baseline and disease-related characteristics in Iranian Parkinson’s disease patients (n = 114)

**Scale/Variable**	**PDQ-39**		**PDQ-8**	
	**Spearman Rho**	**P-value**	**Spearman Rho**	**P-value**
**Age**	.078	0.407	0.024	0.803
**Duration of disease**	0.396	<0.001*	0.342	<0.001*
**UPDRS score**				
Part I-mental	0.572	<0.001*	0.607	<0.001*
Part II-ADL	0.653	<0.001*	0.613	<0.001*
Part III-motor	0.447	<0.001*	0.386	<0.001*
Part IV-complications	0.275	0.003*	0.288	0.002*
Total	0.635	<0.001*	0.591	<0.001*
**Hoehn & Yahr stage**	0.442	<0.001*	0.376	<0.001*
**Schwab & England ADL scale**	-0.598	<0.001*	-0.503	<0.001*
**Daily levodopa dose**	0.313	0.001*	0.270	0.004*
**Duration of levodopa administration**	0.302	0.001*	0.258	0.006*

*Statistical significant correlation (*P* < 0.05).

## Discussion

In most of the previously published studies on HRQoL in PD, the long-form 39-item questionnaire (PDQ-39) is validated in different languages. One paper showed the validity and acceptable reliability of the Persian translated PDQ-39 questionnaire in an Iranian PD population with a Cronbach’s alpha coefficient as high as 0.93 [11]. However, there are few reports on the validity of the short-form version (PDQ-8), none in Persian language, and even less research to compare the psychometric properties of the short-form (PDQ-8) versus the long-form (PDQ-39). In addition, most of the previous projects have used the nested PDQ-8 as part of the original PDQ-39 questionnaire. Using data from both nested and independent PDQ-8, our study was able not only to assess the reliability of the Persian translated PDQ-8, but also to compare the psychometric properties of the nested and independent PDQ-8 with that of the PDQ-39.

In our study, factor analysis confirmed that the PDQ-8 questionnaire is a one-dimensional instrument and this short version of PDQ is not able to measure different dimensions of the original PDQ-39. The Cronbach’s alpha of the entire PDQ-39 was quite high (0.94) in our study, which was in line with a previous report on Iranian PD patients [11]. Reliability coefficient was significantly smaller for the independent shorter version of the questionnaire (PDQ-8) (0.74) compared to the original PDQ-39. This could be expected due to the smaller number of questions in the short-form version which was as low as one fifth. Statistically, the magnitude of the Cronbach’s alpha coefficient is dependent upon the number of items in the instrument and its average inter-item correlation [21]. However, with moderate range of inter-item correlations, higher alpha coefficient is expected with more items and fewer items tend to produce lower estimates of the Cronbach’s alpha [22]. Even for the long-form version, our analysis showed that though all of the PDQ-39 items correlate well with their scale specific total score, dimensions such as “*social support*”, “*cognition*”, “*communication*” and “*bodily discomfort*” had relatively lower total Cronbach’s alpha coefficient ranging from 0.65 to 0.68 compared to other domains with more items. Moreover, the item-to-scale internal consistencies for these domains are also among the lowest coefficients. Similar findings were previously shown in another study on the Chinese translation of the PDQ-39 [23]. The lower reliability index of the abovementioned dimensions could stem from both statistical and cultural reasons. In addition to the fewer number of items, the content of these dimensions are more dependent of the cultural barriers and differences compared to other domains such as “*mobility*” and “*activity of daily living*”.

Internal consistency of the Persian version of the PDQ-8 was shown to be significant for all of the eight items with correlation coefficients ranging between 0.46 and 0.70. No further improvement occurred in alpha coefficient after deletion of any of the single items of the independent PDQ-8. The fact that item 3 on “*emotional well-being*” had the largest effect on reliability of the PDQ-8 demonstrates that mental-related scales affect PDQ-8 scores more prominently than motoric dimensions such as “ADL”. Item specific consistency analysis of the nested PDQ-8 questions showed that item 27 on “*problems with close personal relationships*” in the “*social support*” domain was the only PDQ-8 question to have the highest correlation coefficient with the sum score of the corresponding domain in the original long version PDQ-39.

Table 5 summarizes the results for reliability analysis of the PDQ-8 in different languages and/or cross-cultural studies. The Cronbach’s alpha coefficient was estimated to be lower than 0.9 in all studies (between 0.72 and 0.88), which is almost lower than PDQ-39 in different reports. Our Cronbach’s alpha estimate (0.74) is quite similar to the Greek (0.72) [10], Italian (0.72) [24] and English version in Canadian (0.72) [25] and Singaporean (0.75) [9] populations while some other studies showed higher alpha coefficients [7,26-28]. As shown in Table 5, the item-to-scale internal consistency of the Persian PDQ-8 is within the acceptable range compared to other reports. Generally, the independent Persian version of the PDQ-8 showed acceptable internal consistency, which is in line with the other studies using the independent PDQ-8 [7,28].

**Table 5:** Reliability analysis of the PDQ-8 questionnaire in different cross-cultural studies using different translated versions

**NO.**	**Author, year**	**Sample size**	**Language**	**Cronbach’s alpha**	**Internal consistency (item-to-scale)**
1	**Katsarou et al., 2004**[10]	228	Greek	0.72	-
2	**Martines-Martin et al., 2004**	64 (patient)	Spanish	0.842-0.843	-
		59 (caregiver)			
3	**Tan et al., 2004**[9]	88	English (in Singapore)	0.75	-
4	**Tan et al., 2007**[27]	104 (English)	English and Chinese	0.81	0.44-0.68
		79 (Chinese)		0.87	
5	**Jenkinson & Fitzpatrick, 2007**[7]	812	English (USA), English (Canada), Spanish, Italian, Japanese	0.73-0.88	0.28-0.94
6	**Franchignoni et al., 2008**[24]	200	Italian	0.72	0.24-0.59
7	**Huang et al., 2011**[28]	100	Chinese (Taiwan)	0.81	0.37-0.76
8	**Dal Bello-Haas et al., 2011**[[25]	24	English (Canada)	0.72	-
9	**Present Study, 2013**	114	Persian	0.74	0.46-0.70

Having data from both nested and independent PDQ-8, inter-rater reliability was found to be quite high with an ICC of 0.98 for the entire score of the two versions of the PDQ-8. Katsarou et al. also reported an ICC of 0.72 for the test-retest reliability of summary index of PDQ-8 and PDQ-39 [10]. The Bland-Altman plot also confirmed that the difference between the two scores (independent and nested PDQ-8) was ignorable. Both PDQ-39 and PDQ-8 showed acceptable convergent and criterion validity having almost strong correlations with external measures including PD duration, PD severity assessed by UPDRS, Hoehn and Yahr stage, Schwab and England ADL score, daily cumulative and duration of levodopa medication. Of interest, the first part of UPDRS on “*mentation, behavior, and mood*” is pointed out as the only scale where its correlation was found to be stronger with PDQ-8 than PDQ-39. This shows the acceptable capacity of the short-form PDQ-8 to represent the mental and psychiatric features of PD in the assessment of HRQoL. However, no significant difference was generally found in the validity of the PDQ between the long- and short-form of the questionnaire in correlation with PD-related severity scales. Interestingly, replacement of the selected items in the shorter PDQ with those with the highest internal consistency within each domain in the original PDQ-39 did not show any improvement in the reliability of the PDQ-8 scale. Jenkinson et al reported similar findings that the replacement of some items in the shorter version PDQ-8 eventually decreased the Cronbach’s alpha coefficient of reliability [7].

Having data on both nested and independent PDQ-8, we comprehensively evaluated and compared the psychometric properties of the Persian version of PDQ-8 and PDQ-39 to assess HRQoL in PD patients. However, our study has some limitations. First, there is a possibility of selection bias as the study population selected from an outpatient movement disorder clinic with fewer number of PD patients in severe and/or end-stage of the disease. This might restrict the generalisability of the findings to mainly a mild-to-moderate PD population. Second, both of the long- and short-form questionnaires were completed in one visit, which made it possible to have a memory effect on the answers to the second questionnaire. Consequently, it could result in an overestimation of the reliability indices of the short-form scale.

In conclusion, we found the Persian version of the short-form PDQ (PDQ-8) to be a valid and reliable instrument to assess disease-specific HRQoL in a PD population when used independently. Our findings support the use of Persian-translated PDQ-8 among an Iranian PD population with proper psychometric characteristics. Mostly due to statistical reasons the reliability of the shorter version is lower. However, its validity was found to be almost similar to the original PDQ-39, especially in mental/behavioral domains. Although the PDQ-8 items were not necessarily those with the highest internal consistency in the components of the long-form PDQ-39, they entirely showed an acceptable validity and reliability in the Persian-translated version. Nevertheless, PDQ-8 cannot provide detailed information about different components of the HRQoL as the PDQ-39 does. Yet, PDQ-8 remains a practical and informative instrument in daily clinical practice where clinicians are in shortage of time and when a validated self-administered brief questionnaire is appreciated. Since PDQ-8 has been widely validated in different studies accredited as a general indicator of HRQoL and responsive to treatment effects, it is a suitable research instrument to be used in broad international multi-center clinical trials.

## Competing interest

The authors have neither competing interest to declare in relation to the content of this paper.

## Authors’ contributions

SMF designed the study, carried out data collection, analyzed data and prepared the first and revised version of the manuscript. AR and NN took part in data collection and wrote the first draft of the paper. GAS performed the clinical examination of the patients throughout data collection. AD and JL conceived and designed the study. All authors read and approved the final manuscript.
